# The role of CD68+ macrophage in classical Hodgkin lymphoma patients from Egypt

**DOI:** 10.1186/s13000-019-0912-3

**Published:** 2020-02-04

**Authors:** Osama Mohamed, Ahmed El Bastawisy, Nasr Allahlobi, Mona S. Abdellateif, Abdel Rahman N. Zekri, Sabry Shaarawy, Zeinab Korany, Marwa Mohanad, Abeer A. Bahnassy

**Affiliations:** 1 Department of Medical Oncology, National Cancer Institute, Cairo University, Cairo, 11976 Egypt; 2 Medical Biochemistry and molecular biology, Cancer Biology Department, National Cancer Institute, Cairo University, Cairo, 11976 Egypt; 3 Molecular Virology and Immunology Unit, Cancer Biology Department, National Cancer Institute, Cairo University, Cairo, 11976 Egypt; 4 Biochemistry Department, College of Pharmaceutical Sciences and Drug Manufacturing, Misr University for Science and Technology, 6th October, Cairo, 12945 Egypt; 5 Pathology Department, National Cancer Institute, Cairo University, Cairo, 11976 Egypt

**Keywords:** cHL, TAM, CD68, CD20, Survival, Prognosis

## Abstract

**Background:**

*CD68*+ tumor-associated macrophages (TAM) play an important role in the progression of classical Hodgkin lymphoma (cHL). We assessed the role of *CD20* and *CD68* + TAM in a cohort of cHL patients from Egypt and correlated the number of *CD68* + cells with patients’ characteristics, response to treatment, overall and progression free survival rates (OS & PFS).

**Methods:**

*CD20* expression and *CD68* + TAM numbers were assessed in representative tumor tissues obtained from 81 cHL patients using flowcytometry (FCM), immunohistochemistry (IHC), and Rt-PCR techniques.

**Results:**

The expression levels of CD68 protein by IHC was high in 27 (33.3%), moderate in 15 (18.5%), low in 15 (18.5%), and negative in 24 (29.6%) patients (*p* = 0.13). CD68-mRNA expression was high in 43/81(53.1%), and low in 38(46.9%) patients (*p* = 0.6). The number of *CD68* + TAM (by FCM) was low (< 20 cells) in 42/81 (51.9%), and high (≥20 cells) in 39/81 (48.1%) patients (*p* = 0.74). *CD68* expression (by FCM, IHC& Rt-PCR) associated significantly with poor response to treatment, decreased *CD20* expression, reduced OS and PFS rates (*p* < 0.001 for all). CD68 expression (by Rt-PCR only) associated significantly with advanced disease stage (*p* = 0.04). The age of the patients, high *CD20* expression & high *CD68+* macrophage number were independent prognostic factors for OS (*p*= 0.02, *p* = 0.008 & *p* = 0.009; respectively). However, the age of the patient, high CD20, and high CD68+ macrophage expression (by FCM&IHC) were independent prognostic factors for DFS (*p*. = 0.004, *p*. = 0.01, *p*. = 0.007 and *p*. = 0.01; respectively).

**Conclusion:**

*CD68* + TAM expression (by Rt-PCR, FCM and/or IHC) can identify patients with poor response to treatment and reduced survival rates (OS& PFS). Assessment of *CD68* + positive macrophages by FCM is superior to other methods (Rt-PCR and IHC) as a prognostic factor for DFS and OS rates.

## Introduction

Hodgkin lymphoma (HL), a malignant disease of the lymphatic system, represents about 10% of all diagnosed lymphoma cases, and only 1% of all cancers in the industrial countries **[**1**].** It is the most commonly diagnosed neoplasm in young adults and it has two incidence peaks, one in the third decade of life and the other after the age of 55 **[**2**].** Despite the high cure rate of HL, which is about 90% in the early-stage and 70% in the advanced-stage patients**,** almost 20% of the cases still experience relapsing or refractory disease and eventually progress to death **[**3**,**^4^**].**

Cases of HL cases are classified into two distinct types, the classical HL (cHL), which is diagnosed in more than 95% of the cases, and the nodular lymphocyte predominant HL (NLPHL) which constitutes the remaining 5% of the cases **[**5**].** The cHL is further subdivided histologically into four subtypes: the nodular sclerosis, which is the commonest subtype (representing 70% of all diagnosed cases), followed by the mixed cellularity in about 20–25%, the lymphocyte-rich in 5%, and the lymphocyte-depleted subtype in only 1% of the cases **[**5**].** These subtypes are characterized by the presence of Hodgkin/Reed-Sternberg (HRS) cells, which are large mono-nucleated or multi-nucleated cells with prominent nucleoli **[**6**].** Although HRS cells represent only 1–2% of the tumor burden, they are considered the key marker for HL diagnosis, and they play an important role in the interaction with other immune-modulatory cells in the tumor microenvironment of HL **[**1**,**^7^**].** It has been established that HRS cells of cHL originate from the germinal center (GC) B-cells **[**8**].** They secret a variety of cytokines and chemokines, including *IL-4, IL-5, IL-10, CCL22* and *CCL5*, which attract the T helper-2 (Th2) and the T-reg cells **[**9**,**^10^**].** Therefore, these cells are considered a driving force for the abnormal immune response, which is commonly reported in the cHL patients. They are also responsible for the presence of a large number of reactive inflammatory cells in the tumor microenvironment including B&T cells, macrophages, eosinophils and mast cells **[**7**].** The neoplastic cells also secrete *TNF-α* and *TGF-β*, which promote the activation of fibroblasts **[**11**],** and the production of collagen type IV in the tumor microenvironment. Type IV collagen reacts with the discoidin domain receptor 1 (*DDR1***)** on the RS cells supporting their survival and proliferation, and hence promoting resistance to treatment and tumor progression **[**12**,**^13^**].**

The tumor microenvironment has a critical role in the development and progression of tumors and it also modifies the clinical outcome of patients with hematological malignancies, especially those with follicular lymphoma and cHL **[**14**,**^15^**].** However, the genomic profile of the HRS cells is still not well- characterized yet due to the relative paucity of these cells within the bulk of the tumor **[**16**].** This has also provoked more studies in the area of tumor microenvironment, especially the TAM and their possible diagnostic, prognostic and predictive values.

Another important issue related to HL is that until now the clinical and laboratory parameters used for the diagnosis of cHL do not permit accurate identification of patients with less favorable clinical outcomes **[**17**].** However, recent studies provided evidence that an increased number of tumor infiltrating macrophages is significantly associated with shortened survival in HL patients and about 20% will have relapse and/or refractory disease associated with increasing late toxic effects. Accordingly, the authors concluded that, the number of the infiltrating TAM should be considered a powerful prognostic predictor in patients with HL **[**18**]**.

Based on these data, the aim of the current study was to assess the role of *CD68+* tumor associated macrophages (TAM) in the development and progression of cHL. This will be achieved via 1) assessment of the number of *CD68* positive macrophages in tissue biopsies, 2) the expression levels of *CD68* and CD20 proteins (by IHC) in tumor samples, and **3)** the expression level of *CD68*-mRNA by Rt-PCR. The emerging data of TAM number, ***CD68*** and ***CD20*** proteins expressions will be correlated to relevant clinico-pathological features of the patients, response to treatment and survival rates [overall survival (OS) and progression free survival (DFS**)].**

## Methods

### Patients

This retrospective cohort study included 81 patients with pathologically confirmed classic Hodgkins lymphoma (cHL) who were diagnosed and treated at the National Cancer Institute (NCI), Cairo University, during the period from 2006 to 2013. The inclusion criteria of the patients were as follows; an age ≥ 18 years with ECOG performance status ≤2 and no other concurrent or previous malignancies. All patients have adequate bone marrow function (WBC count ≥3.0 × 10^9^/L, ANC ≥1.5 × 10^9^ /L, platelet count ≥100 × 10^9^/L, hemoglobin level ≥ 9 g/L), adequate liver and kidney functions, and ejection fraction ≥50%. Patients, who had second malignancy, previously received systemic therapy regimens or if they currently enrolled in another running clinical trial were excluded.

All patients were subjected to complete medical history and physical examination, assessment of vital signs, and ECOG performance status according to World Health Organization (WHO) scale of performance status before and during treatment **[**19**].** They were also subjected to **1)** full laboratory investigations including complete blood count with differential LDH, ESR, and full biochemical panel including liver and renal function tests. **2)** Bone marrow aspiration and representative tumor biopsy, and **3)** radiological evaluation (including CT chest, abdomen& pelvis and echocardiography). All patients were treated according to the NCI and the NCCN guidelines. Evaluation of response to treatment was done according to the Revised Response Criteria for malignant lymphoma **[**20**]**.

### Samples preparation

***CD68*** and ***CD20*** protein expressions were assessed in all tested cases (81) compared to 20 normal lymph nodes samples obtained from patients with reactive hyperplasia. From each formalin fixed, paraffin-embedded tissue (FFPETs) block (patients& control), three sections were obtained onto three positive charged slides (Fisher). One slide was stained with haematoxylin and eosin to confirm diagnosis and assess tumor to normal tissue ratio in the sample (s). The second slide was used to assess the expression level of *CD68* protein by IHC, and the third slide was used to assess the expression levels of *CD20* positive cells by IHC. Only samples including ≥75% neoplastic cells in the sections were analyzed. Another five (5-10 μ) thick sections were cut onto 2 ml, plastic Epindorf tubes for assessment of *CD68+* TAMs by FCM.

### Assessment of *CD68* and *CD20* protein expression by IHC

Slides were de-paraffinized in xylene followed by a series of graded ethanol. Antigen retrieval was done by 2 min pressure-cooking in citrate buffer (pH 6.0), endogenous peroxidases were blocked with 0.3% H_2_O_2_, and non-specific binding was blocked with normal goat serum. Cells were then reacted with the primary *CD68* antibody (mouse anti- human CD68, 1:40, Abcam, MA, USA, ab955) and *CD20* antibody (mouse anti- human CD20, Abcam, MA, USA, ab88247) for 24 h at 4 °C. The secondary antibody (EnVision System/HRP, Dako, Tokyo, Japan) was applied for 1 h, tissue sections were washed with PBS, colored with DAB, counterstained with hematoxylin and examined microscopically. Two cases of cHL, known to be positive for *CD20* and *CD68* were used in each run as positive controls. The negative control was obtained by omitting the primary antibody. The expression levels of either *CD68* or *CD20* were considered low if < 20%, moderate if > 20–50% and high if more than 50% of the neoplastic cells.

### Assessment of *CD68 +* TAM by FCM

According *to Leers MP* et al**.[**21**],** 50 μ thick sections (5–8 sections) of each tumor or normal tissue sample were cut onto 2 ml, sterile, plastic Eppindorph tubes, deparafinized in xylene and rehydrated in a descending series of ethanol. Sections were then immersed in a cold citrate solution (2 mg Citric acid/ ml aqua distilled water, pH = 6.0) and placed in a water path at 80 °C for 2 h. After 15 min cooling period, the sections were rinsed in PBS and digested in a solution of 1 mg trypsin (Sigma, St. Louis, MO) and 1 mg CaCl_2_ in 1 ml TRIS-buffered saline (PH = 7.6) for 5 min at 37 °C. Samples were then filtrated through a 50 μm nylon mesh filter and the cell suspension was centrifuged at 400 g. The pellets were suspended in 1% BSA/PBS buffer and stained with CD68-FITC monoclonal antibody (# 11–0689-42, eBioscience™, US) according to the manufacturer’s instructions. Samples were acquired in the FCM (Becton& Dickinson, BD FACS Calibur), and analyzed using the cell quest software. The FCM results were expressed in comparison to the control samples as follows: low expression if the positive cell number is < 20 and high expression if the positive cell number is ≥20 cells**.**

### Detection of *CD68* by reverse transcriptase PCR (Rt-PCR)

RNA was extracted from FFPET samples of HL and pooled normal lymph nodes (as a control) using RNeasy Mini Kit (Qiagen, Milan, Italy). Retro-transcription was performed using iScriptTM cDNA Synthesis Kit (Bio-Rad, Milano, Italy) according to manufacturer’s instructions. The RT-PCR analysis was performed in 25 μl final volume of with a 10 μl cDNA, 400 nM of each primer for the respective genes according to manufacturer’s instructions using AB-Applied Biosystem, 7500 Fast PCR. The primer sequences were as follow: *CD68:* F: GCTTTGCAATCTCCCTGTTG& R: TTGATCCGGGTTCTTACCTG) and *β-actin* (F:ACAGAGCCTCGCCTTTGC;R: GCGGCGATA TCATCATCC). The cycling conditions were as follow: 50 °C for 2 min and 95 °C for 10 min, followed by 40 cycles at 95 °C for 15 s and 60 °C for 1 min. All samples were assessed in duplicate**.**

### Statistical analysis

All statistical tests were performed using a statistical software package (SPSS Inc. version 22.0; Chicago, IL, USA). The association between CD68 and CD20 expression with relevant clinico-pathological characteristics and response to treatment was assessed with chi-squared or Fischer exact test when appropriate. Association with survival was analyzed using Kaplan-Meier plot and log-rank test. Univariate and multivariate survival analysis was done using the Cox proportional hazard model. All p. values were two-tailed. *p* value < 0.05 was considered statistically significant.

## Results

### Patients’ characteristics

The current study included 51 males (63%) and 30 females (37%) with a mean age of 40.7 ± 15.5 years. Twenty-eight (34.6%) patients presented with early stage (I& II) and 53 (65.4%) presented with advanced disease stage (III&IV). The most common pathological subtype was the nodular sclerosis (45 cases; 55.5%) followed by mixed cellularity (33 cases; 40.7%), lymphocyte- rich (2 cases; 2.46%) and lymphocyte-depletion (1 case; 1.23%). Twenty-two patients (27.1%) presented with constitutional B symptoms, 17 (21%) with Bulky disease ≥10 cm, and 19 (23.4%) had high international prognostic score (IPS) ≥4. All patients were treated according to NCI protocols with ABVD (Adriamycin, bleomycin, vinblastine, dacarbazine) chemotherapy and 20 (24.6%) patients received bleomycin, etoposide, doxorubicin, cyclophosphamide, vincristine, procabazine and prednisone (BEACOPP) as a second line treatment, 9 (9.87%) patients received Involved Field radiotherapy (IFRT), and only one patient (1.6%) underwent bone marrow transplantation **(**Table 1**)**.

**Table 1:** Clinico-pathologic features of the Hodgkin lymphoma patients

Characteristics	Number 81 (%)
Age (years)	
Mean ± SD	40.7 ± 15.5
Gender	
Male	51 (63%)
Female	30 (37%)
Stage	
I	1 (1.23%)
II	27 (33.3%)
III	39 (48.15%)
IV	14 (17.3%)
Histological subtypes	
Nodular sclerosis	45 (55.5%)
Mixed cellularity	33 (40.7%)
Lymphocyte-rich	2 (2.46%)
Lymphocyte- depleted	1 (1.23%)
B symptoms	
Negative	59 (72.8%)
Positive	22 (27.1%)
Bulky disease	
≥ 10CM	17 (21%)
< 10CM	64 (79%)
International Prognostic Score (IPS)	
≥ 4 (high risk)	19 (23.4%)
< 4 (low risk)	62 (76.5%)
Management	
Primary treatment ABVD	81(100)
IFRT	8 (9.87%)
2nd line	20 (24.6%)
BMT	1 (1.2%)

*IFRT* Involved Field radiotherapy, *BMT* bone marrow transplantation

### Assessment of *CD68* and *CD20* protein expression by IHC

**Fig. 1:**
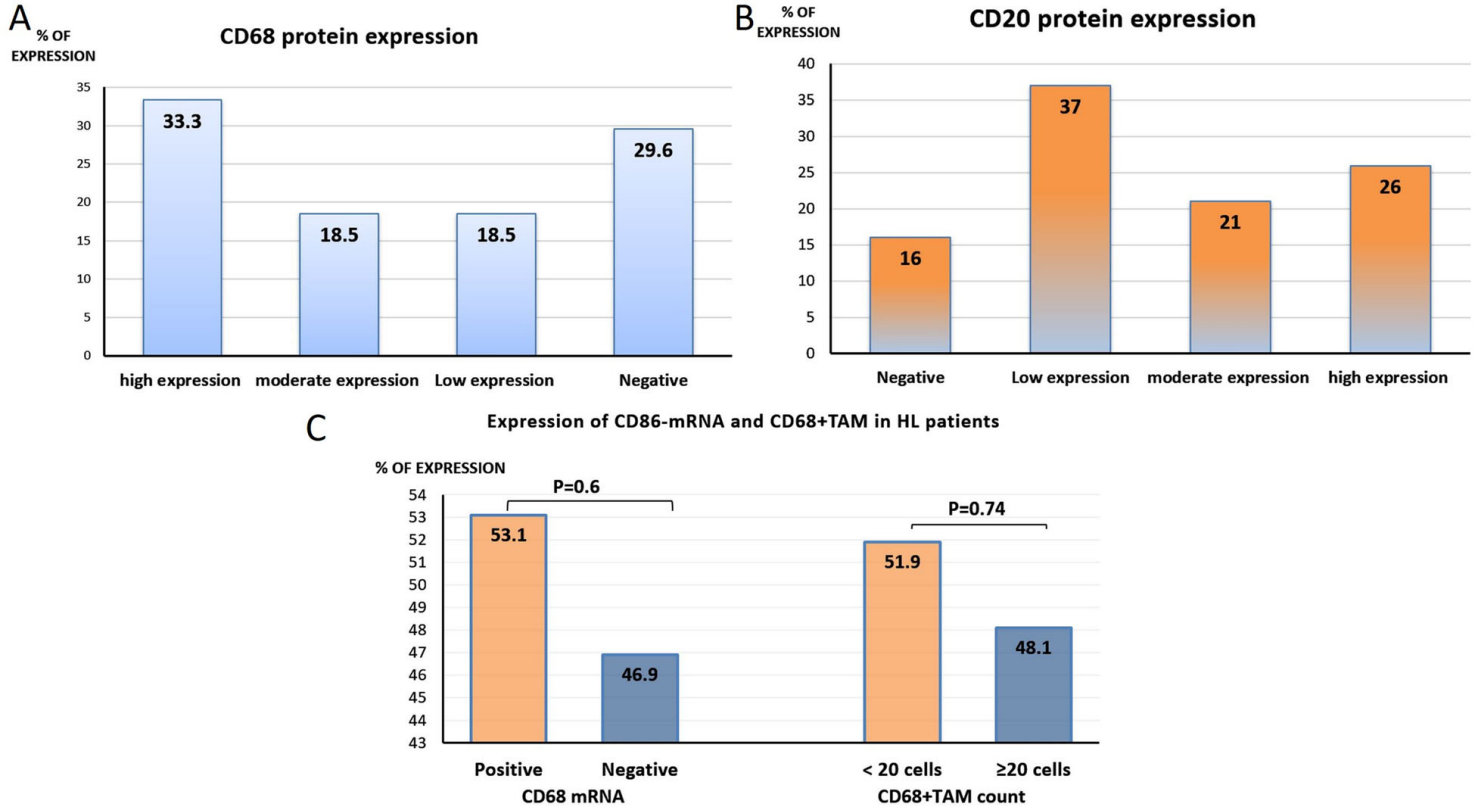
the expression levels of **a**) CD68 proteins by IHC, **b**) CD20 proteins by IHC, **c**) CD68 by FCM and Rt-PCR

**Fig. 2:**
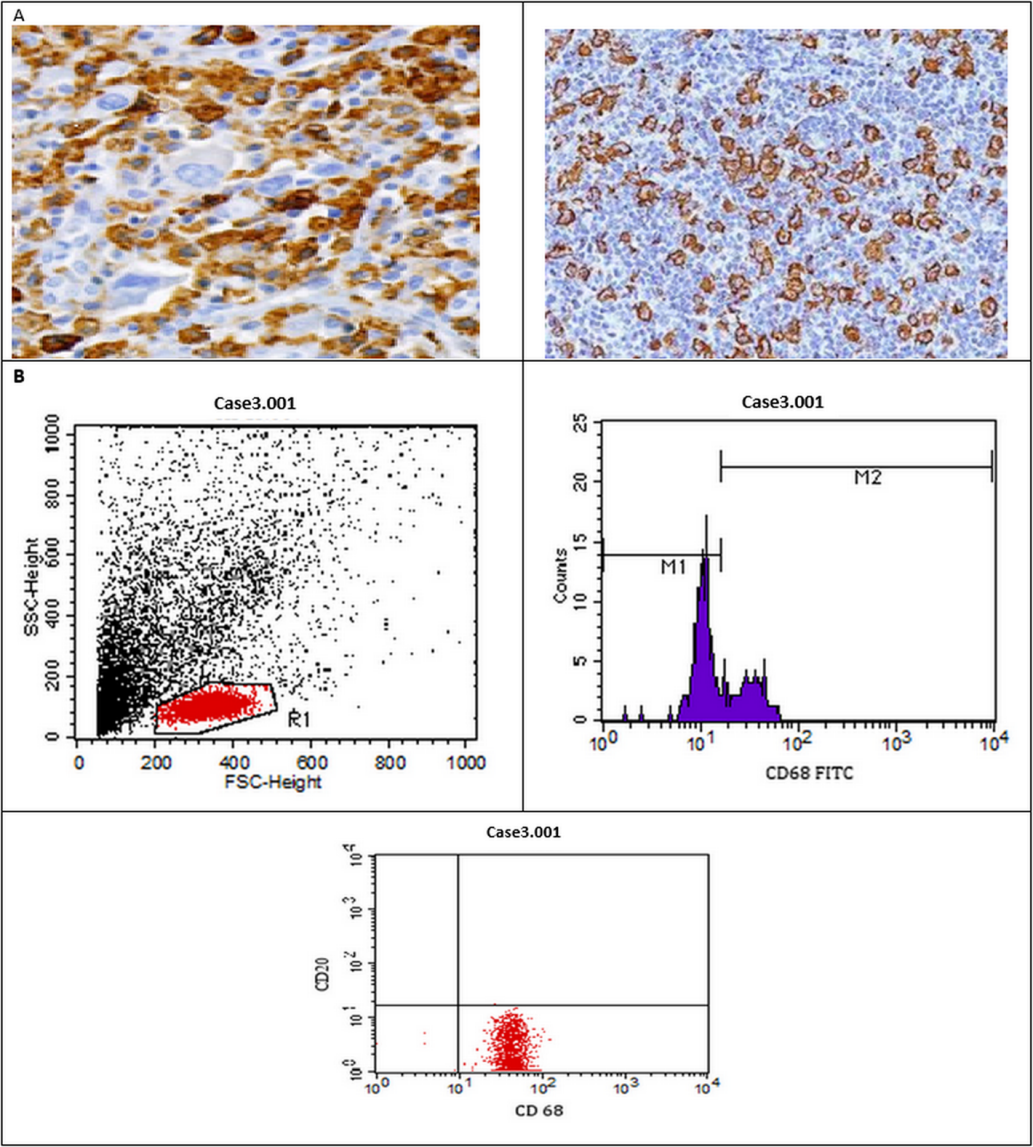
**a** Immuno-histochemical staining of CD68 + TAM in cHL (200X), **b** Assessment of CD 68 and CD 20 by flow cytometry

fifty-seven patients (70.4%) were positive for *CD68* protein expression by IHC, 15 (18.5%) of them showed low expression, another 15 (18.5%) showed moderate expression and 27 (33.3%) patients showed high expression. On the other hand, 24 patients (29.6%) were negative for *CD68* protein expression (*p* = 0.13, Fig. 1a**).** As for, *CD20* protein expression was detected in 68 patients, of which 30(37.0%) showed low *CD20*- expression, 17(21.0%) showed moderate expression, and 21(26.0%) showed high expression. Thirteen patients (16%) were completely negative for *CD20*- expression (*p* = 0.049, Fig. 1b & Fig. 2a**).**

### Assessment of *CD68* + TAM cells by FCM

Cases were considered positive for *CD68 +* TAM if the *CD68+* cells exceeded the number of positive cells in the normal control group (> 20 cells). Accordingly, out of the 81 patients assessed 42 (51.9%) have a low number of *CD68* + TAM (< 20 cells), and 39 (48.1%) have a high number of *CD68 +* TAM (≥20 cells) in the assessed HL patients. The difference between the two groups was statistically non-significant (*p* = 0.74, Fig. 1c & Fig. 2b**)**.

### Assessment of *CD68* mRNA expression

The *CD86* mRNA was overexpressed in 43/81 (53.1%) patients, and normally expressed in 38/81 (46.9%) patients compared to the control group (*p* = 0.6, Fig. 1c**).**

### Correlations between *CD68* expression and relevant clinico-pathological features of the patients

**Fig. 3:**
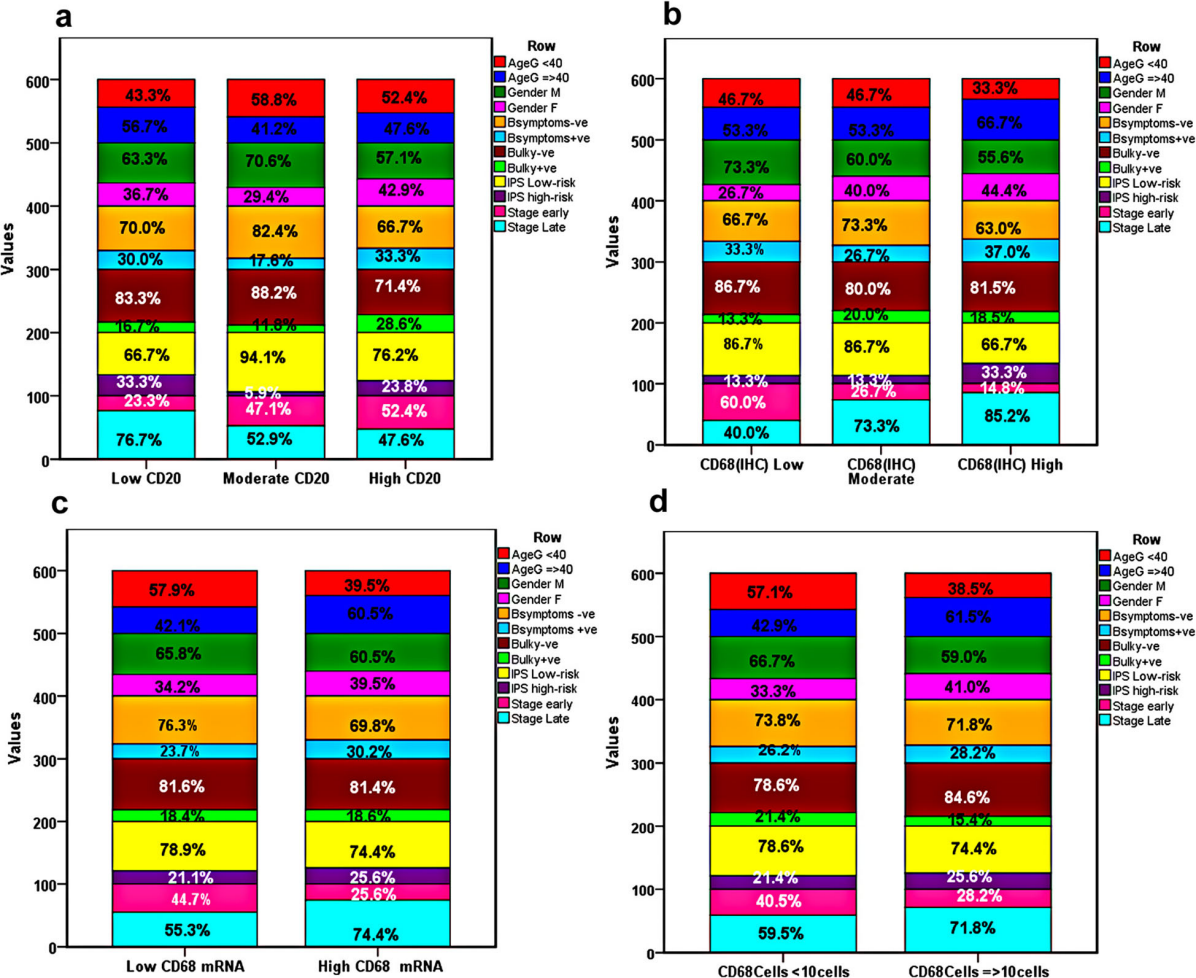
Correlation between the clinic-pathological features of the patients and **a**) CD20 protein expression, **b**) CD68 expression by IHC, **c**) CD68 mRNA by Rt-PCR, and **d**) CD68 + TAM by FCM

There was statistically significant association between *CD68* mRNA expression and advanced disease stage, since out of all patients expressing *CD68 mRNA* (43), 30 (69.8%) were disease stage III&IV compared to 13 (30.2%) were stage I&II (*p* = 0.04). Similarly, *CD20* expression associated significantly with tumor stage, since in all late stage disease patients (42), 23(76.7%) patients showed *CD20-*low expression, 9 (52.9%) showed moderated expression, and 10 (47.6%) patients showed *CD20-*high expression (*p* = 0.048). The expression levels of *CD68* protein, measured by IHC associated with advanced tumor stage, however this association was of borderline significance (*p* = 0.07). on the other hand, no significant association was found between *CD68* expression (either by IHC, FCM, or Rt-PCR) and age, gender, pathological subtypes, B symptoms, IPS, bulky disease, or in all tested cases (*p* > 0.05, Table 2& Table 3**,** Fig. 3**).**

**Table 2:** Correlation between CD68 (IHC and mRNA) expression and clinic-pathological features of the patients

Characteristics	CD68 protein n(%)			*P* value^a^	*CD68* (mRNA) (*n* = 43)	*P* value^b^
	Low (*n* = 15)	Moderate (*n* = 15)	High (*n* = 27)		n (%)	
Age						
< 40	7(46.7)	7(46.7)	9(33.3)	0.13	17(39.5)	0.1
≥ 40	8(53.3)	8(53.3)	18(67.7)		26(60.5)	
Gender						
Male	11(73.3)	9(60.0)	15(55.6)	0.68	26(60.5)	0.62
Female	4(26.7)	6(40.0)	12(44.4)		17(39.5)	
Stage						
I-II	9(60.0)	6(40.0)	6(22.2)	0.07	13(30.2)	0.04*
III-IV	6(40.0)	9(60.0)	21(77.8)		30(69.8)	
B-symptoms						
Negative	10(66.7)	11(73.3)	17(63.0)	0.24	30(69.8)	0.51
Positive	5(33.3)	4(26.7)	10(37.0)		13(30.2)	
Bulky disease						
Negative	13(86.7)	12(80.0)	22(81.5)	0.95	35(81.4)	0.98
Positive	2(13.3)	3(20.0)	5(18.5)		8(18.6)	
IPS						
Low risk <4	13(86.7)	13(86.7)	18(66.7)	0.36	32(74.4)	0.63
High risk **≥**4	2(13.3)	2(13.3)	9(33.3)		11(25.6)	
Pathology						
Mixed cellularity	4(26.7)	6(40.0)	12(44.4)	0.15	18(41.9)	0.39
Nodular necrosis	11(73.3)	7(46.7)	15(55.6)		23(53.5)	
Lymphocyte-rich	0(0.0)	2(13.3)	0(0.0)		2(4.7)	
Lymphocyte-depleted	0(0.0)	0(0.0)	0(0.0)		0(0.0)	

^a^Chi-square test was used, ^b^ Fischer exact test was used, *Significant at *p* < 0.05

**Table 3:** Association between CD68+ cell count, CD20 expression and patients’ cinico-pathological features

Characteristics	CD20 protein n(%)			*P* value^a^	CD68 cell count n(%)		*P* value^b^
	Low (*n* = 30)	Moderate (*n* = 17)	High (*n* = 21)		< 20 (42)	≥20(39)	
Age							
< 40	13(43.3)	10(58.8)	11(52.4)	0.64	24(57.1)	15(38.5)	0.09
≥ 40	17(56.7)	7(41.2)	10(47.6)		18(42.9)	24(61.5)	
Gender							
Male	19(63.3)	12(70.6)	12(57.1)	0.86	28(66.7)	23(59.0)	0.47
Female	11(36.7)	5(29.4)	9(42.9)		14(33.3)	16(41.0)	
Stage							
I-II	7(23.3)	8(47.1)	11(52.4)	0.048*	17(40.5)	11(28.2)	0.25
III-IV	23(76.7)	9(52.9)	10(47.6)		25(59.5)	28(71.8)	
B-symptoms							
Negative	21(70.0)	14(82.4)	14(66.7)	0.70	31(73.8)	28(71.8)	0.84
Positive	9(30.0)	3(17.6)	7(33.3)		11(26.2)	11(28.2)	
Bulky disease							
Negative	25(83.3)	15(88.2)	15(71.4)	0.56	33(78.6)	33(84.6)	0.48
Positive	5(6.7)	2(11.8)	6(28.6)		9(21.4)	6(15.4)	
IPS							
Low risk< 4	20(66.7)	16(94.1)	16(76.2)	0.21	33(78.6)	29(74.4)	0.66
High risk **≥**4	10(33.3)	1(5.9)	5(23.8)		9(21.4)	10(25.6)	
Pathology							
Mixed cellularity	11(36.7)	8(47.1)	9(42.9)	0.55	15(35.7)	18(46.2)	0.63
Nodular necrosis	17(56.7)	8(47.1)	12(57.1)		25(59.5)	20(51.3)	
Lymphocyte-rich	2(6.7)	0(0.0)	0(0.0)		1(2.4)	1(2.6)	
Lymphocyte-depleted	0(0.0)	1(5.9)	(0.0)		1(2.4)	0(0.0)

### Overall response to treatment

Out of 81 patients assessed, 61 (75.3%) showed good clinical response [complete remission (CR) and partial remission (PR)] compared to 20 (24.7%) non-responders [stable disease (SD) and progressive disease (PD)]. Poor response to treatment was observed in patients more than 40 years old compared to those less than 40 years old [5/20 (25%) versus 15/20 (75.0%); respectively, *p* = 0.017). In addition, Patients with early disease stage showed better response to treatment compared to those with advanced disease stage. In patients with poor response to treatment (20 patients); 16 (80%) were tumor stage III or IV, compared to four patients (20%) only with early disease stage II or I (*p* = 0.03).

Significant association was also found between complete clinical response to treatment and *CD20* expression since all patients with high *CD20* expression (21/61; 34.4%) showed good response to treatment (*p* < 0.001). On the other hand, poor response to treatment was observed in patients with high *CD86* protein and/or mRNA expression. In the in non-responding group 80% (16/20) of the patients had high *CD68* protein expression, and 90% (18/20) expressed *CD86+* mRNA transcript (*p* < 0.001). Similarly, a high number of *CD86+* macrophage (> 20 cells) was present in patients with poor response to treatment 17/20 (85%) compared to 3/20 (15%) in patients with *CD86 +* cells less than 20 cells (*p* < 0.001). in contrast, no statistically significant association was present between patients’ response to treatment and any of the relevant clinicopathological features of the patients including gender, B symptoms, IPS or a bulky disease (*p* > 0.05, Table 4).

**Table 4:** Patients’ response to treatment

	Response		*P* value*
	Non-Responding (20) (SD,PD)	Responding (61) (CR,PR)	
Age			
< 40 (39)	5(25.0%)	34(55.7%)	**0.017**^**a**^
≥ 40 (42)	15(75.0%)	27(44.3%)	
Gender			
Male (51)	11(55.0%)	40(65.6%)	0.40 ^**a**^
Female (30)	9(45.0%)	21(34.4%)	
B-symptoms			
Negative (59)	15(75.0%)	44(72.1%)	0.80 ^**a**^
Positive (22)	5(25.0%)	17(27.9%)	
Bulky disease			
Negative (66)	16(80.0%)	50(82.0%)	0.84 ^**a**^
Positive (15)	4(20.0%)	11(18.0%)	
IPS			
Low risk (62)	15(75.0%)	47(77.0%)	0.85 ^**a**^
High risk (19)	5(25.0%)	14(23.0%)	
Stage			
Early (33)	4(20.0%)	29(47.5%)	**0.03**^**a**^
Advanced (48)	16(80.0%)	32(52.5%)	
CD20-IHC			
Negative (13)	7(35.0%)	6(9.8%)	
Low-expression (30)	12(60.0)	18(29.5%)	**< 0.001**^**b**^
Moderate-expression (17)	1(5.0%)	16(26.2%)	
High-expression (21)	0(0.0%)	21(34.4%)	
*CD68*-mRNA			
Negative (38)	2(10.0%)	36(59.0)	**< 0.001**^**a**^
Positive (43)	18(90.0%)	25(41.0)	
CD68-IHC			
Negative (24)	1(5.0%)	23(37.7%)	**< 0.001**^**b**^
Low-expression (15)	0(0.0%)	15(24.6%)	
Moderate-expression (15)	3(15.0%)	12(19.7%)	
High-expression (27)	16(80.0%)	11(18.0%)	
CD68 cell count			
< 20 cells	3(15.0%)	39(63.9%)	**< 0.001**^**a**^
≥ 20 cells	17(85.0%)	22(36.1%)	

^a^Fischer exact test was used, ^b^Chi-square test was used, *Significant at *p* < 0.05PD: progressive disease, *SD* Stable disease, *CR* Complete remission, *PR* Partial Remission

### Progression free survival (DFS)

**Fig. 4:**
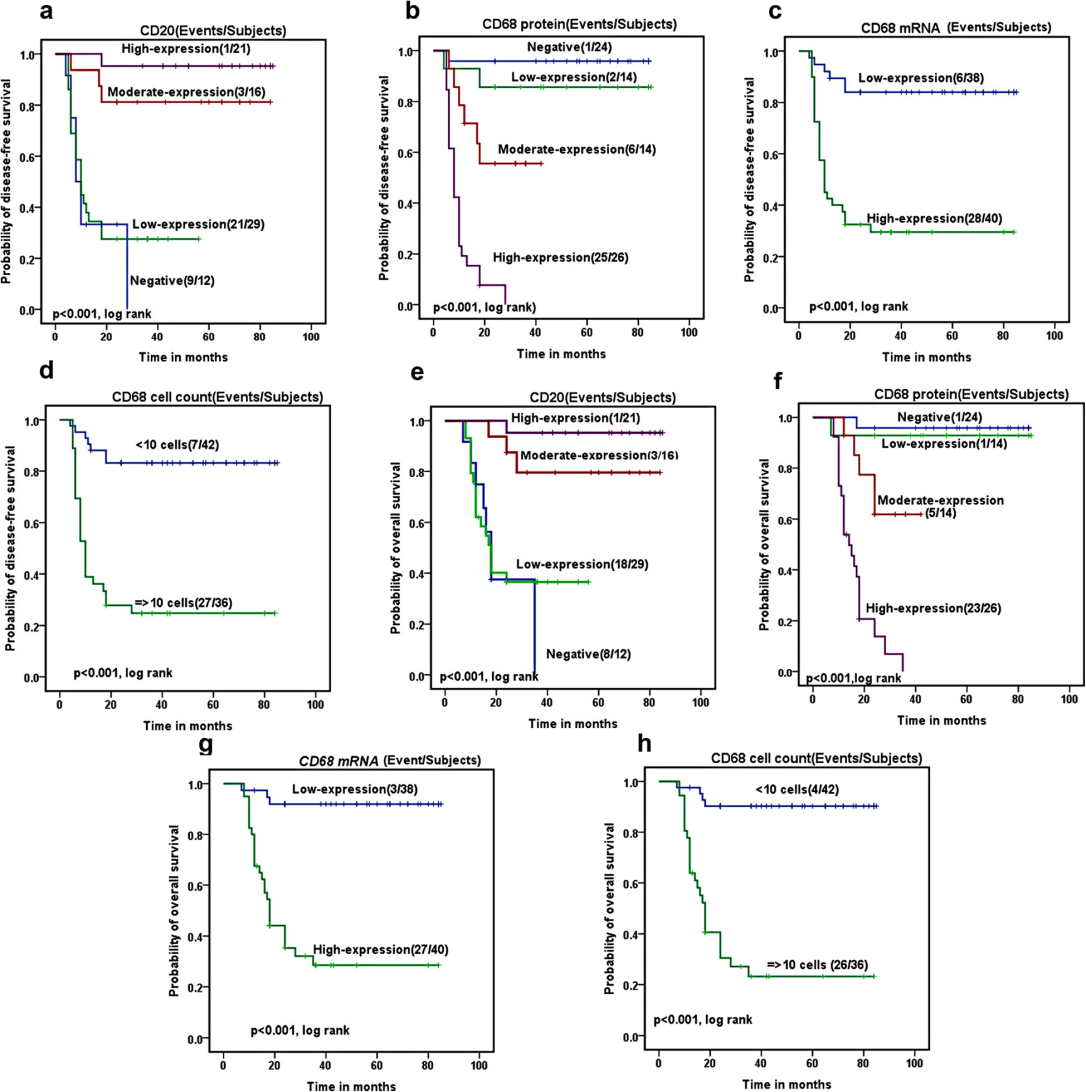
correlation of the disease free survival (DFS) and **a**) CD20 protein expression, **b**) CD68 expression by IHC, **c**) CD68 mRNA by Rt-PCR, and **d**) CD68 + TAM by FCM. Correlation of the overall survival (OS) with **e**) CD20, **f**) CD68 expression by IHC, **g**) CD68 mRNA by Rt-PCR, and h) CD68 + TAM by FCM

The five years DFS rate for all HL patients was 32.5%. It associated significantly with patients age (72.1% in patients ≤40 versus 41.5% in patients ≥40 years, *p* = 0.003), and disease stage (68.6% in early stage compared to 47.6% in advanced stage *p* = 0.03). *CD68* protein expression measured by IHC associated significantly with reduced DFS rates (85.7, 55.6 and 0%) in patients with low, moderate and high expressions; respectively, compared to 95.8% in patients who did not express *CD68* protein (*p* < 0.00). Similarly, *CD68* mRNA expression associated significantly with reduced DFS rates (84.1% in *CD68 mRNA* negative patients compared to 29.5% in *CD68* mRNA positive patients, *p* < 0.001). Patients with decreased number of CD68+ macrophage (< 20 cell) had a higher DFS rates compared to those with increased number (> 20 cells) of CD68+ macrophage (83.2 and 24.7% respectively, *p* < 0.00). improved DFS rate associated significantly with increased *CD20* expression (27.6, 81.3 and 95.2%) in patients with low, moderate and high expression respectively, compared to 0% in patients with *CD20* negative expression (*p* < 0.001, Fig. 4a-d).

No significant association was detected between DFS rates and any the other clinico-pathological feature of the patients assessed including gender, B symptoms, IPS, bulky disease and tumor pathology (*p*. > 0.05, Additional file 1: Table S1).

### Overall survival rates (OS)

The 5 years OS for the assessed patients was 60.1%. It associated significantly with the age of the patients (71.4% in patients ≤40 versus 50% in patients ≥40 years, *p* = 0.03) and disease stage (74.0% in early stage compared to 50.4% in advanced stage, *p* = 0.015). It also associated significantly with *CD68* protein expression assessed by IHC (92.9% in patients with low expression, 61.9% moderate expression and 0.0% in high expression compared to 95.8% in patients negative for *CD68* protein (*p* < 0.001). As for *CD86* mRNA expression, the OS rates were 92% of *CD68 mRNA* negative patients compared to 28.5% of *CD68* mRNA positive patients (*p* < 0.001). Similarly, Patients with decreased number of CD68+ macrophage (< 20 cell) had a higher OS rates compared to those with increased number (> 20 cells) of CD68+ macrophage (90.3 and 23.2% respectively, *p* < 0.001). Moreover, improved OS rates associated significantly with increased *CD20* expression (36.5, 79.5 and 95.2%) in patients with low, moderate and high expression respectively, compared to 0% in patients with *CD20* negative expression (*p* < 0.001, Fig. 4e-h).

No significant association was found between the OS rate and any of the other relevant clinico-pathological features of the patients including gender, B symptoms, IPS, bulky disease, and tumor pathology (*p* > 0.05, Additional file 1: Table S2).

### Multivariate analysis for OS and DFS rates

On multivariate analysis, the age of the patients, *CD20* expression and the number of *CD68+* macrophage by FCM were independent prognostic factors for OS (*p* = 0.02, *p* = 0.008 & *p* = 0.009; respectively), whereas the age of the patient, *CD20* expression, *CD68+* macrophage expression (by FCM and IHC) were independent prognostic factors for DFS. The *p* values were 0.004, 0.01, 0.007 and *p* = 0.01; respectively. *CD68 mRNA* expression was not an independent prognostic factor neither for DFS nor for OS (*p* = 0.07 and *p* = 0.40; respectively, Table 5**).**

**Table 5:** univariate and multivariate analysis for OS and DFS of the patients

Factors	Overall survival (OS)			Disease-free survival (DFS)		
	HR	95%CI	*p*-value	HR	95%CI	*p*-value
Univariate analysis						
Age (yrs) < 40 vs ≥40	2.2	1.0–4.8	**0.038**	2.8	1.3–5.9	**0.006**
B symptoms -ve vs + ve	1.1	0.5–2.4	0.78	1.23	0.6–2.5	0.58
Bulky disease + ve vs -ve	1.8	0.6–5.1	0.28	0.61	0.24–1.6	0.31
IPS Low vs High	1.9	0.9–4.0	0.10	1.5	0.7–3.2	0.26
Gender M vs F	1.2	0.6–2.5	0.59	1.23	0.6–2.5	0.51
Pathology	0.9	0.5–1.6	0.73	0.86	0.49–1.5	0.61
Stage early vs late	2.6	1.2–5.8	**0.02**	2.2	1.0–4.5	**0.04**
CD20 -ve vs + ve	0.15	0.05–0.29	**< 0.001**	0.15	0.07–0.33	**< 0.001**
*CD68 (mRNA)* –ve vs + ve	13.2	3.9–43.7	**< 0.001**	6.6	2.7–16.0	**< 0.001**
CD68 (IHC) –ve vs + ve	4.7	2.6–8.2	**< 0.001**	4.0	2.4–6.6	**< 0.001**
CD68 CC < 10 vs ≥10 cells	12.2	4.2–32.3	**< 0.001**	7.1	3.1–16.4	**< 0.001**
Multivariate analysis						
Age (yrs) < 40 vs ≥40	2.5	1.1–5.6	**0.02**	3.2	1.5–7.0	**0.004**
Stage early vs late	2.2	0.8–6.1	0.11	1.8	0.69–4.5	0.23
CD20 -ve vs + ve	0.24	0.09–0.69	**0.008**	0.29	0.11–0.74	**0.01**
*CD68* (mRNA)–ve vs + ve	2.6	0.28–24.6	0.40	5.8	0.90–32.0	0.07
CD68 (IHC) –ve vs + ve	5.2	0.65–41.3	01.2	8.2	1.6–41.8	**0.01**
CD68+ cells (FCM) < 20 vs ≥20 cells	9.5	1.8–51.5	**0.009**	7.7	1.7–33.7	**0.007**

### Correlation between CD68+ cell counts and the expression levels of *CD68* mRNA, CD68 protein and CD20

Significant correlations were present between the *CD68*+ cell count, ***CD68*** mRNA and protein expressions (*p* < 0.001, for all). The *CD20* expression protein correlated inversely with the expression levels of *CD68* protein (r = − 594, *p* < 0.001), *CD68 mRNA* (r = − 0.417, *p* < 0.001), and *CD68+* cell count (r = − 0.387, *p* = 0.001, Table 6**)**.

**Table 6:** Correlation of the CD68 cell count and expression of *CD68* mRNA, CD68 protein and CD20 in patients with LH

	CD68(IHC)	CD68 mRNA	CD68 cell count	CD20 (IHC)
CD68(IHC)	–	*r* = 0.804** *p* < 0.001	*r* = 732** *p* < 0.001	*r* = −594** *p* < 0.001
*CD68* mRNA	*r* = 0.804** *p* < 0.001	–	*r* = 0.856** *p* < 0.001	*r* = −0.417** *p* < 0.001
CD68+ cell count	*r* = 732** *p* < 0.001	*r* = 0.856 *p* < 0.001	–	*r* = − 0.387** *p* = 0.001
CD20 (IHC)	*r* = − 594** *p* < 0.001	*r* = − 0.417** *p* < 0.001	*r* = − 0.387** *p* = 0.001	–

**Correlation is highly significant at *p* < 0.01

## Discussion

A unique feature of HL is that the neoplastic cells crucially depend on the supporting microenvironment and its cellular composition, particularly TAM which affect the tumor prognosis. However, the role of TAM in adult classical Hodgkin lymphoma (cHL) patients remains controversial, and there is a great debate in the literature about its prognostic and diagnostic significance. In the current study, we assessed the expression levels of CD68 + TAM in HL patients by different techniques including IHC for CD68 protein expression, Rt-PCR for CD68 mRNA transcript, and FCM for evaluation of the number of CD68+ TAM, to detect the most reliable and sensitive methods for assessment of patients with HL from Egypt.

The current study provide an evidence that CD68 expression (by Rt-PCR, FCM and/or IHC) associated significantly with reduced DFS and OS rates of the assessed HL patients. In addition, our data also revealed that DFS and OS rates associated significantly with patients’ age, advanced disease stage and reduced CD20 protein expression. These data are in concordance with recent published studies [22–24], reported that intratumoral CD68 macrophage infiltration and B cell markers (CD20) expression can accurately predict DFS and OS rates in the HL patients. Kamper et al. [25], also reported that High numbers of CD68+ and CD163+ macrophages in cHL associated significantly with worse OS through their correlation with the presence of Epstein–Barr virus (EBV) in the neoplastic cells which in turn, lead to worse outcomes mainly in older individuals [26]. Another study done by Gotti et al., [17] demonstrated that a proportion of tumor-infiltrating macrophages (by IHC) greater than 25% is associated with unfavorable clinical outcome and shorter DFS in 106 early stage cHL patients from Italy. These data are contradictory to that observed by Azambuja et al. [27]**,** who found that there was no significant association between CD86+ /or CD163+ TAM (by IHC) and the relevant clinico-pathological features of the patients, OS and DFS rates in 265 well characterized cHL patients from USA. Similarly another two studies done by Agur et al., and Kayal et al. [28, 29]**,** reported that CD86+ macrophage did not associate significantly with the baseline characteristics of the cHL patients, response to treatment and DFS.

Furthermore, better response to treatment was observed in patients with reduced CD68 expression (by Rt-PCR, FCM and/or IHC), age lower than 40 years old, patients presented with early disease stage, and in those who were positive for CD20 protein expression. Our data in this context are in agreement with some previous reports including the study of Cuccaro et al. [30], who demonstrated that the evaluation of the number of CD68 positive cells and B-symptoms at diagnosis could help to categorize low-risk patients regardless of the positive interim PET. They reported also that CD68 positive cells and B-symptoms were strong predictors for DFS in the assessed HL patients from Italy. However, according to our data, B-symptoms did not significantly affect clinical response to treatment, OS or DFS rates. Thus, the evaluation of CD68+ TAM at diagnosis could predict patients’ clinical outcome more accurately than other factors.

Our data showed that the expression levels of CD68 (by Rt-PCR, FCM and/or IHC) associated significantly with the poor prognostic factors of cHL patients including the decreased B cell markers (CD20) expression, and only CD68 mRNA expression associated significantly with advanced disease stage. There was no significant association with other relevant clinic-pathological features including age, gender, histological subtype, B symptoms, IPS, and bulky disease. However, Koh et al., [22] who conducted another study on cHL patients from Korea and demonstrated that CD68+ TAM associated significantly with gender and high risk IPS in cHL patients. This discrepancy in the results might be attributed to the differences in the sample size, ethnic and etiological variations among the patients included in the two studies. Finally, our results confirm the data reported by Guo et al., who performed a meta-analysis study on 22 eligible studies containing a total number of 2959 patients, they reported that high density of CD68+ TAMs in the tumor microenvironment of adult cHL could predict poor OS, shorter DFS, and advanced disease stage [31].

In multivariate analysis, the expression levels of CD20 and the age of the patients were independent prognostic factors for both DFS and OS rates. Regarding the expression of CD68, its expressions by FCM and/or IHC were independent prognostic factors for OS, while only its expression by FCM was independent prognostic factors for DFS. These data are in concordance with many recent studies [30, 32] reported that the number of CD68+ macrophages (by IHC) outperformed the international prognostic score (IPS) in adult cHL by multivariate analysis. Similarly, Koh et al. observed that CD68 (by IHC) was independent prognostic factor for DFS, and it was not an independent prognostic marker for OS in cHL patients [23].

## Conclusions

Based on our data, we conclude that CD68 + TAM expression by either Rt-PCR, FCM and/or IHC could significantly identify patients with poor response to treatment, reduced OS and PFS rates. However, the assessment of number of CD68 positive macrophages by FCM is more superior to the other methods (Rt-PCR and IHC), as an independent prognostic factors for both DFS and OS rates by multivariate analysis. In addition, only CD68 mRNA expression associated significantly with advanced disease stage of the patients. Thus, if we used other methods such as FCM or RT-PCR for assessment of CD68 + TAM could increase its significance rather than the detection of CD68 by IHC. These data, when verified on a larger study including a higher number of patients (confirmatory set) will open a new avenue for anticancer-targeted therapy against these cells.

## Supplementary information

**Additional file 1: Table S1.** association between Disease free survival (DFS) and relevant clinic-pathological features of the patients. **Table S2.** association between overall survival rates (OS) and relevant clinic-pathological features of the patients.

## Data Availability

All data generated or analyzed during this study are included in this published article [and its supplementary information files].

## Abbreviations

ABVD: Adriamycin, bleomycin, vinblastine, dacarbazine
BEACOPP: Bleomycin, etoposide, doxorubicin, cyclophosphamide, vincristine, procabazine and prednisone
CHL: Classical Hodgkin lymphoma
DDR1: Discoidin domain receptor 1
DFS: Progression free survival
FCM: Flowcytoetry
FFPETs: Formalin fixed, paraffin-embedded tissue
HRS: Hodgkin/Reed-Sternberg
IFTR: Involved Field radiotherapy
IHC: Immunohistochemistry
IPS: International prognostic score
NCI: National cancer institute
NLPHL: Nodular lymphocyte predominant HL
OS: Overall free survival
TAM: Tumor-associated macrophages
TH2: T helper-2
WHO: World Health Organization
