# Supplementary material for: The role of CD68+ macrophage in classical Hodgkin lymphoma patients from Egypt
Source: Diagn Pathol. 2020 Feb 4;15:10. doi: 10.1186/s13000-019-0912-3 (PMC7001371; doi:10.1186/s13000-019-0912-3)
Supplement: Supplementary file 1 — Additional file 1: Table S1. association between Disease free survival (DFS) and relevant clinic-pathological features of the patients. Table S2. association between overall survival rates (OS) and relevant clinic-pathological features of the patients. [file 13000_2019_912_MOESM1_ESM.docx]

**Supp. 1:** Progression free survival (PFS) and clinic-pathological features of the patients

| **Factors** | **Progression free survival %** | | | | | **Median (Ms)**  **(95%CI)** | **P value** |
| --- | --- | --- | --- | --- | --- | --- | --- |
|  | **6 months** | **1yr** | **2yrs** | **3yrs** | **≥5yrs** |  |  |
| **All (78)** | 98.7 | 85.2 | 56.3 | 39.3 | 32.5 | 28.4  (20-36.9) |  |
| **Age (yrs)** |  |  |  |  |  |  | **0.003** |
| <40 (37) | 97.3 | 78.4 | 75.6 | 72.1 | 72.1 | NA |  |
| ≥40 (41) | 70.7 | 53.7 | 41.5 | 41.5 | 41.5 | 17.0  (9.2-24.8) |  |
| **B symptoms** |  |  |  |  |  |  | 0.57 |
| Negative (56) | 82.1 | 67.9 | 60.5 | 58.4 | 58.4 | NA |  |
| Positive (22) | 86.4 | 59.1 | 50.0 | 50.0 | 50.0 | 18.0 (NA) |  |
| **Bulky disease** |  |  |  |  |  |  | 0.29 |
| Negative (63) | 79.4 | 61.9 | 55.4 | 53.5 | 53.5 | NA |  |
| Positive (15) | 100.0 | 80.0 | 66.7 | 66.7 | 66.7 | NA |  |
| **IPS** |  |  |  |  |  |  | 0.24 |
| Low risk (59) | 86.4 | 69.5 | 60.8 | 58.8 | 58.8 | NA |  |
| High risk (19) | 73.7 | 52.6 | 47.4 | 47.4 | 47.7 | 13.0 (NA) |  |
| **Gender** |  |  |  |  |  |  | 0.50 |
| Male (50) | 86.0 | 76.0 | 60.0 | 60.0 | 60.0 | NA |  |
| Female (28) | 78.6 | 64.1 | 52.8 | 49.0 | 49.0 | 28.0 (NA) |  |
| **Pathology** |  |  |  |  |  |  | 0.58 |
| Mixed cellularity (31) | 77.4 | 71.0 | 58.1 | 58.1 | 58.1 | NA |  |
| Nodular necrosis (44) | 86.4 | 59.1 | 54.4 | 51.5 | 51.5 | NA |  |
| Lymphocyte-rich (2) | 100.0 | 100.0 | 100.0 | 100.0 | 100.0 | NA |  |
| Lymphocyte-depleted (1) | 100.0 | 100.0 | 100.0 | 100.0 | 100.0 | NA |  |
| **Stage** |  |  |  |  |  |  | **0.03** |
| Early (27) | 84.0 | 81.3 | 71.9 | 68.6 | 68.6 | NA |  |
| Advanced (51) | 76.1 | 54.3 | 49.8 | 47.6 | 47.6 | 17.0  (NA) |  |
| **CD20-IHC** |  |  |  |  |  |  |  |
| Negative (12) | 75.0 | 33.3 | 0.0 | 0.0 | 0.0 | 8.0(5.3-10.7) |  |
| Low-expression (29) | 69.0 | 37.9 | 27.6 | 27.6 | 27.6 | 10.0(7.4-12.6) |  |
| Moderate-expression (16) | 93.8 | 93.8 | 81.3 | 81.3 | 81.3 | NA | <0.001 |
| High-expression (21) | 100.0 | 100.0 | 95.2 | 95.2 | 95.2 | NA |  |
| ***CD68*-mRNA** |  |  |  |  |  |  |  |
| Negative (38) | 94.7 | 89.5 | 84.1 | 84.1 | 84.1 | NA | <0.001 |
| Positive (40) | 72.5 | 42.5 | 32.5 | 29.5 | 29.5 | 10.0(7.5-12.5) |  |
| **CD68-IHC** |  |  |  |  |  |  |  |
| Negative (24) | 95.8 | 95.8 | 95.8 | 95.8 | 95.8 | NA |  |
| Low-expression (14) | 92.9 | 92.9 | 85.7 | 85.7 | 85.7 | NA | <0.001 |
| Moderate-expression (14) | 92.9 | 71.4 | 55.6 | 55.6 | 55.6 | NA |  |
| High-expression (26) | 61.5 | 19.2 | 7.7 | 0.0 | 0.0 | 8.0(6.0-9.9) |  |
| **CD68 cell count** |  |  |  |  |  |  |  |
| <20 cells(42) | 95.2 | 88.1 | 83.2 | 83.2 | 83.2 | NA | <0.001 |
| ≥20 cells(36) | 69.4 | 38.9 | 27.8 | 24.7 | 24.7 | 10.0(7.9-12.1) |  |

**Supp. 2**: Overall survival rate (OS) and clinic-pathological features of the patients

| **Factors** | **Overall survival %** | | | | | **Median (Ms)**  **(95%CI)** | **P value** |
| --- | --- | --- | --- | --- | --- | --- | --- |
|  | **6 months** | **1yr** | **2yrs** | **3yrs** | **≥5yrs** |  |  |
| **All (78)** | 96.2 | 82.1 | 63.4 | 60.1 | 60.1 | NA |  |
| **Age (yrs)** |  |  |  |  |  |  | **0.03** |
| <40 (37) | 100.0 | 94.6 | 74.9 | 71.4 | 71.4 | NA |  |
| ≥40 (41) | 100.0 | 70.7 | 52.9 | 50.0 | 50.0 | 28.0  (NA) |  |
| **B symptoms** |  |  |  |  |  |  | 0.77 |
| Negative (56) | 100.0 | 83.9 | 65.3 | 60.9 | 60.9 | NA |  |
| Positive (22) | 100.0 | 77.3 | 58.3 | 58.3 | 58.3 | NA |  |
| **Bulky disease** |  |  |  |  |  |  | 0.27 |
| Negative (63) | 100.0 | 77.8 | 61.5 | 57.6 | 57.6 | NA |  |
| Positive (15) | 100.0 | 100.0 | 70.1 | 70.1 | 70.1 | NA |  |
| **IPS** |  |  |  |  |  |  | 0.09 |
| Low risk (59) | 100.0 | 86.4 | 69.0 | 64.6 | 64.6 | NA |  |
| High risk (19) | 100.0 | 68.4 | 45.6 | 45.6 | 45.6 | 24.0 (NA) |  |
| **Gender** |  |  |  |  |  |  | 0.58 |
| Male (50) | 100.0 | 82.0 | 63.8 | 63.8 | 63.8 | NA |  |
| Female (28) | 100.0 | 82.1 | 62.6 | 54.2 | 54.2 | (NA) |  |
| **Pathology** |  |  |  |  |  |  | 0.61 |
| Mixed cellularity (31) | 100.0 | 77.4 | 63.6 | 63.6 | 63.6 | NA |  |
| Nodular necrosis (44) | 100.0 | 84.1 | 60.6 | 54.6 | 54.6 | NA |  |
| Lymphocyte-rich (2) | 100.0 | 100.0 | 100.0 | 100.0 | 100.0 | NA |  |
| Lymphocyte-depleted (1) | 100.0 | 100.0 | 100.0 | 100.0 | 100.0 | NA |  |
| **Stage** |  |  |  |  |  |  | **0.015** |
| Early (27) | 100.0 | 93.8 | 81.3 | 74.0 | 74.0 | NA |  |
| Advanced (51) | 100.0 | 73.9 | 50.4 | 50.4 | 50.4 | NA |  |
| **CD20-IHC** |  |  |  |  |  |  |  |
| Negative (12) | 100.0 | 75.0 | 37.5 | 0.0 | 0.0 | 18.0(14.9-21.4) | <0.001 |
| Low-expression (29) | 100.0 | 62.1 | 36.5 | 36.5 | 36.5 | 18.0(14.0-22.0) |  |
| Moderate-expression (16) | 100.0 | 100.0 | 87.5 | 79.5 | 79.5 | NA |  |
| High-expression (21) | 100.0 | 100.0 | 95.2 | 95.2 | 95.2 | NA |  |
| ***CD68*-mRNA** |  |  |  |  |  |  |  |
| Negative (38) | 100.0 | 97.4 | 92.0 | 92.0 | 92.0 | NA | <0.001 |
| Positive (40) | 100.0 | 67.5 | 35.3 | 28.5 | 28.5 | 18.0(15.6-20.4) |  |
| **CD68-IHC** |  |  |  |  |  |  |  |
| Negative (24) | 100.0 | 100.0 | 95.8 | 95.8 | 95.8 | NA |  |
| Low-expression (14) | 100.0 | 92.9 | 92.9 | 92.9 | 92.9 | NA | <0.001 |
| Moderate-expression (14) | 100.0 | 92.9 | 61.9 | 61.9 | 61.9 | NA |  |
| High-expression (26) | 100.0 | 53.8 | 13.8 | 0.0 | 0.0 | 14.0(10.5-17.4) |  |
| **CD68 cell count** |  |  |  |  |  |  |  |
| <20 cells (42) | 100.0 | 97.6 | 90.3 | 90.3 | 90.3 | NA | <0.001 |
| ≥20 cells(36) | 100.0 | 63.9 | 30.5 | 23.2 | 23.2 | 18.0(15.7-20.2) |  |
